# Sun Basking in Red Wood Ants *Formica polyctena* (Hymenoptera, Formicidae): Individual Behaviour and Temperature-Dependent Respiration Rates

**DOI:** 10.1371/journal.pone.0170570

**Published:** 2017-01-23

**Authors:** Štěpánka Kadochová, Jan Frouz, Flavio Roces

**Affiliations:** 1 Department of Behavioural Physiology and Sociobiology, Biocenter, University of Würzburg, Würzburg, Germany; 2 Department of Ecology, Faculty of Science, Charles University, Prague, Czech Republic; 3 Institute for Environmental Studies, Faculty of Science, Charles University, Prague, Czech Republic; Universite Francois-Rabelais de Tours, FRANCE

## Abstract

In early spring, red wood ants *Formica polyctena* are often observed clustering on the nest surface in large numbers basking in the sun. It has been hypothesized that sun-basking behaviour may contribute to nest heating because of both heat carriage into the nest by sun-basking workers, and catabolic heat production from the mobilization of the workers’ lipid reserves. We investigated sun-basking behaviour in laboratory colonies of *F*. *polyctena* exposed to an artificial heat source. Observations on identified individuals revealed that not all ants bask in the sun. Sun-basking and non-sun-basking workers did not differ in body size nor in respiration rates. The number of sun-basking ants and the number of their visits to the hot spot depended on the temperature of both the air and the hot spot. To investigate whether sun basking leads to a physiological activation linked with increased lipolysis, we measured respiration rates of individual workers as a function of temperature, and compared respiration rates of sun-basking workers before and two days after they were allowed to expose themselves to a heat source over 10 days, at self-determined intervals. As expected for ectothermic animals, respiration rates increased with increasing temperatures in the range 5 to 35°C. However, the respiration rates of sun-basking workers measured two days after a long-term exposure to the heat source were similar to those before sun basking, providing no evidence for a sustained increase of the basal metabolic rates after prolonged sun basking. Based on our measurements, we argue that self-heating of the nest mound in early spring has therefore to rely on alternative heat sources, and speculate that physical transport of heat in the ant bodies may have a significant effect.

## 1. Introduction

Wood ants of the genus *Formica* belong to those social insect species whose colonies are able to keep relatively stable temperatures inside the nest [1–4], as for instance honey bees [5], fungus-growing termites [6], some bumble bees [7], stingless bees [8] and leaf-cutting ants [9]. For the control of climate conditions inside the nest, large nests with good insulation properties are crucial [10]. Metabolic heat produced by ant workers [3, 11] or by associated microorganisms [12, 13, 14] is supposed to be an important inner source of heat. The level of nest thermoregulation depends on many factors other than size, e.g., population size, moisture and thermal conductivity of the nest material [4].

Temperature of a *F*. *polyctena* nest is usually higher and more stable than ambient temperature throughout the whole year [2, 3, 15]. During the period of ant activity and brood production, which averages approximately 100 days [16, 17], ants maintain an area in their nest where the temperature is stable and does not drop below 20°C; this place is called the heat core [2, 15, 18]. High temperatures in spring are required for the development of the sexual brood and for oviposition in queens [16]. In winter, nest temperature changes with ambient temperature, but temperatures in the hibernation chambers remain stable at 1–2°C [3, 18].

The main outer source of heat for wood ant nests is solar radiation. The physical structure of the ant mound is supposed to work as a solar collector [19–21]. Moreover, solar energy may increase the metabolism of ant workers because of the increase in body temperature [22, 23], and it also helps to keep the nest material dry [2], thus increasing nest insulation. Thermal properties of the nest material, mainly thermal conductivity and heat storage capacity, which are tightly linked to nest moisture, may influence solar energy income and heat distribution throughout the nest [10].

Solar radiation can be also absorbed by the bodies of ants located on the nest surface, and heat was hypothesized to be brought into the nest by returning ants that sun basked [24]. Ant bodies are dark and contain a large proportion of water (ca. 75% in wood ants, unpublished results), with a high specific heat capacity. This make the ant bodies an ideal medium for heat carriage. In early spring, ant workers form large clusters on the mound surface and bask in the sun. At approximately the same time a rapid increase of inner nest temperature is observed [2, 3, 24]. Based on such indirect evidence, it has been hypothesized that those ants that sun bask on the nest surface increase their body temperature and later move into the nest, where both the absorbed heat and that catabolically produced because of the workers’ higher metabolic rate are passively lost and warm the nest interior [3, 22, 24]. More recent laboratory measurements of cooling rates and behaviour of sun-basking ants strongly support this hypothesis [25]. At 10°C, for instance, workers with body temperatures of 25°C were observed to lose the absorbed heat only after 2 minutes, within which they were able to walk over more than 50 cm [25].

Clustering at the nest surface may contribute to a quick increase of body temperatures of sun-basking ants because workers inside a cluster will have lower thermal losses than single workers [4]. It has been suggested that heat transport by workers returning from the outside, where they exposed themselves to the sun, may work all around the year, even without clustering [2, 17]. It has also been argued that sun-basking behaviour can have one additional effect, namely, a sustained mobilization of the workers’ lipid reserves, i.e., an increased rate of lipolysis [11]. Sun basking is more pronounced, and it occurs earlier, in replete workers, i.e., those workers that accumulated large lipid reserves before the winter and use them to raise the sexual brood in early spring [11, 26, 27].

During the weeks when sun basking is observed, workers show a marked rate of lipid and carbohydrate catabolism, with fat body lipids representing the largest energy storage of workers [26]. Sun basking over 8 days, in addition, was shown to trigger the development of the labial glands in replete workers in early spring [28], the secretions of which are used to feed the sexual brood. It is therefore likely that sun-basking behaviour may also serve as a trigger of lipid catabolism, leading to a sustained increase in the respiration rate and accordingly to an increased metabolic heat production in sun-basking workers as compared to non-sun basking workers. In addition to a potentially sustained high metabolic rate triggered by sun basking, workers may produce additional heat because of the higher body temperatures they reach after sun basking, which may also result in more intense locomotion.

In the 70´s, sun basking was described for *Formica* ants in the laboratory under a heat source, in groups of 500–1000 workers [29]. It was observed that workers aggregated and sun basked under the artificial heat source, creating clusters in which they moved „circularly” up and down, like honey bees moving in a winter cluster. First, workers were sun basking on the surface, then trying to enter the cluster to avoid overheating, and then coming to the surface again [29]. The authors found that the number of workers sun basking increased with increasing temperature of the heat source and water availability.

The present experiments were inspired by the studies described above [25, 29]. We investigated sun-basking behaviour of *F*. *polyctena* ants under laboratory conditions, in colonies exposed to an artificial heat source that simulates a hot spot. Thanks to the use of modern technologies we were able to observe and quantify interindividual variability in sun-basking behaviour in workers. We aimed at answering the following questions: Do all ants take part in sun-basking behaviour? Are there differences among workers in the time they spend on the hot spot and in the number of visits? Is the number of visits to the hot spot related to the duration of the single visits? Do sun-basking and non-sun-basking ants differ morphologically?

In addition, we explored the hypothesis that sun basking may contribute to self-heating of the nest in early spring due to the activation of the workers’ lipid reserves and the resulting high, sustained metabolic heat production [11, 22]. To address this question, we measured first the respiration rates of individual workers as a function of temperature, to characterize the temperature dependence of respiration across the ranges of temperatures ants experience after overwintering. Second, we compared respiration rates of non-sun-basking workers with those of sun-basking workers before and two days after they were allowed to expose themselves to a heat source over 10 days, at self-determined intervals, to highlight a putative long-lasting increase in respiration rates triggered by sun basking.

## 2. Materials and Methods

### 2.1. Ant keeping

One queen-right colony of *Formica polyctena*, with a nest size of *ca*. 10 liters, was collected in the field nearby the town Sulzbach am Main, Germany (49° 54' 42.417" N 9° 9' 23.394" E) in 2011. This species is protected under the Convention on International Trade in Endangered Species of Wild Fauna and Flora (CITES). Permit for collection and maintenance of a wood ant colony in the laboratory for scientific research was issued by the Unterfranken government (Regierung von Unterfranken, Würzburg, Germany) in 2011. The colony was split in two large queen-right groups (henceforth called colonies S1 and S2) and placed in plastic nest boxes (100x100x80cm), which were kept in an incubator at 25°C over 8 months from April to the end of November, and at an overwintering temperature of 5°C from December to March. The switch between 25°C and 5°C took place over 12 days, following 5°-step changes every 3 days. The nest boxes were maintained at 50% relative humidity, under a 12h:12h dark-light cycle.

For the experiments, ants were taken from hibernation in the middle of February. The nests were moved to a temperature-controlled chamber at 10°C (a temperature that approximates the air temperature experienced by field nests in spring [2, 3, 15, 17, 18], and the light-dark cycle was kept at 12h:12h. The colonies were provided with *ad libitum* water, a honey-water solution (*ca*. 33% sugar) and dead crickets every other day. For the experiments, a subset of 100 workers, called subcolony, was separated from one of the colonies and placed into a nest box (9x19x9cm), together with a handful of nest material. Experimental subcolonies were provided daily with *ad libitum* water and honey-water solution. All experiments were performed at the Biocenter, University of Würzburg, from February to May in the year 2014.

### 2.2. General experimental setup

**Fig 1:**
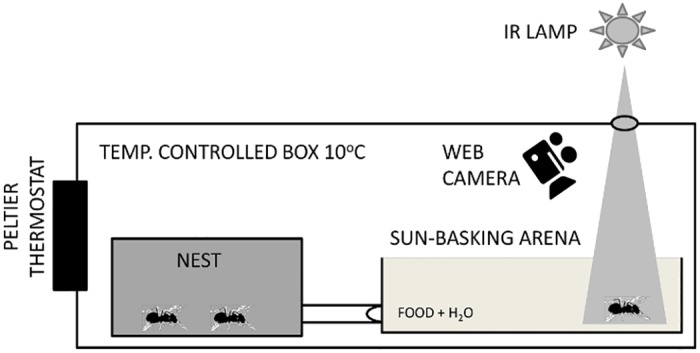
Experimental setup for recording of sun-basking behaviour. For a detailed description see part 2.2.

A nest box containing a subcolony was placed inside an experimental box (45x75x30cm) with controlled temperature (10°C) and isolated from the surroundings. The nest was connected to an open arena (20x20cm) via a short tube, where foraging and sun basking took place (Fig 1). In one corner of the open arena honey-water was offered, whilst in the other nonadjacent corner the hot spot was offered for four hours each consecutive experimental day. The nest box was placed inside the experimental box at least 3 days prior to the experiments, to allow ants to habituate and to explore the arena.

The basic schedule for the sun-basking experiments was as follows: A single experiment run over 10 days. Each day an “artificial sun”–an infrared lamp of 150W - was presented in the box for 4 hours, creating a hot spot on the floor of the sun-basking arena, approximately in the middle of the light phase of the light-dark cycle. We used infrared light to investigate the ant reactions to the presence of a heat source, and to avoid reactions to visible light. The lamp was placed 15cm above the experimental box, and the heat was allowed to enter the arena through a small opening (4cm in diameter) in the box lid (Fig 1). The aggregation of ants was video-recorded for 2 hours, one hour after the heat source was switched on, i.e., in the middle of the period in which the hot spot was available. In each experiment, 20 hours of sun-basking behaviour were therefore video-recorded over the 10 days. A digital camera (Logitech HD Webcam c525) was used for video recording, and the videos were saved in Windows Media-Audio-/Videodata format (.wmv) into a mass storage device.

**Fig 2:**
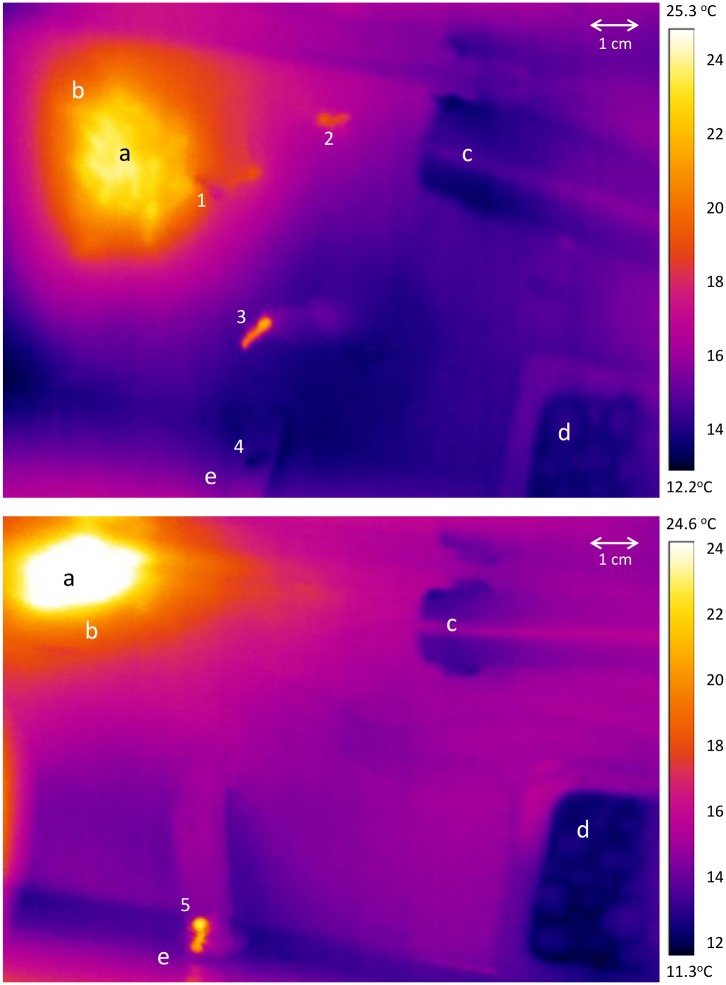
Thermal images of sun basking ants in the experimental arena obtained with an infrared thermographic camera (Flir P620). Top image: A dense cluster of sun-basking ants (a: white to yellow colours) on the hot spot (b: red colour) is visible in the upper left corner; (c): water source; (d) food patch; (e) nest entrance. Behaviour of individual ants: (1): cold worker entering the sun cluster; (2): warm worker walking towards the water source; (3): warm worker walking towards the nest; (4) cold worker leaving the nest. Lower image: (a)-(e): as in the top image; (5): warm worker walking over the ramp leading to the nest entrance. On the lower image, the warm area on the left side and the warm background represent an artifact generated by a nearby digital web cam.

At the end of each daily sun-basking period, an IR-photograph of the cluster of sun-basking ants was obtained with an infrared thermographic camera (Flir P620), from which the temperatures of both the hot spot and the cluster of sun-basking ants were obtained (Fig 2). For the temperature of the hot spot, the whole circular area illuminated by the IR lamp was taken in account (Fig 2b), and the average temperature of this circle was recorded using the FLIR tools. For the ant body temperature, we recorded the maximal temperature in the middle of the ant cluster (Fig 2a). The emissivity of the ant bodies was set at 0.99 for calibration, which is the value used for other social insects such as bees and wasps [30].

### 2.3. Sun-basking experiments

#### Experiment 1: Group colour marking on a daily basis

For the experiment 1, ants from colony S1 were taken. To identify ants that sun basked over consecutive days, different colour marks were used on a daily basis. The basic setup common for all experiments was as described above. After 2 hours video recording of sun-basking behaviour, i.e., 3 hours after the hot spot was made available, all ants previously sitting on the hot spot were collected with forceps and marked with a colour spot on the gaster (or on the thorax if there was no more free space on the gaster over the days of the experiment), using harmless water-resistant colours (Edding 750).

Each day an additional colour dot was added to those ants that already participated in sun baking the previous day. Every day, the number of ants with colour marks from previous days and the individual colour combination of each marked ant worker were recorded. After the marking procedure, ants were released back to the open arena. Mortality was counted daily as dead ants were carried by nestmates and dropped outside the nest. At the end of the experiment, all living ants were examined for colour marks and the number of those that took part in sun basking (i.e., ants with at least one colour dot) and did not (i.e., without colours) were counted.

#### Experiments 2 and 3: Individual tagging

**Fig 3:**
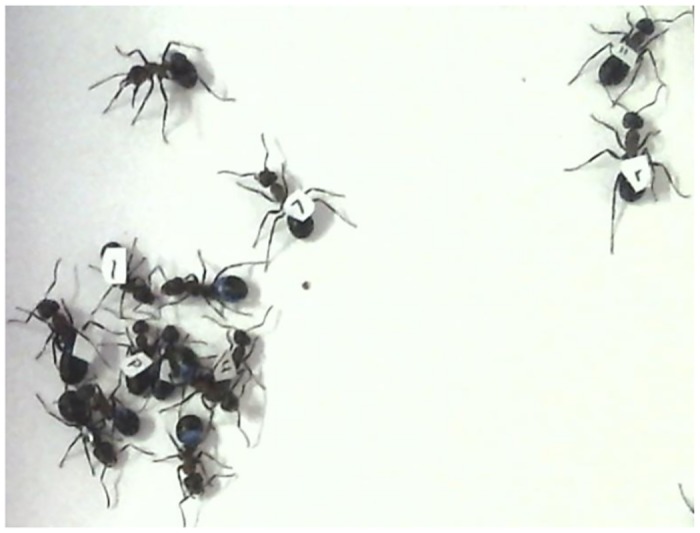
Group of ants with individual paper tags glued on their gasters. A snapshot from video recording. Individuals coded as “7”, “r”, “~“, “H”, “p”, “h” and unmarked individuals are clearly visible. Note that several ants have lost their paper tag so their identification was not possible.

In experiment 1 we observed several ants that repeatedly visited the hot spot, which were therefore called “sun-basking workers”, and others that never visited the hot spot, which were called “non-sun-basking workers”. To gain a closer insight into the behaviour of individual ants on the hot spot, in the next experiments we individually marked 100 ants with a small paper tag with an unique symbol (Fig 3) glued on the gaster with a drop of Edding colour, following a marking technique developed in our laboratory by S. Mildner [31]. Two experiments using individual paper tags were performed, which involved ants from the different colonies: ants from colony S1 in experiment 2, and ants from colony S2 in experiment 3. The basic setup was the same as described for experiment 1, with the difference that marking with paper tags was done prior to the experiment. Therefore, there was no manipulation of ants during the whole experimental period and ants were not disturbed after the end of their sun-basking period.

### 2.4. Data collection and analysis

Video recordings were observed in commonly accessible players (Windows media player, VLC) and ant behaviour was analysed using the program Observer (TheObserver, Version 2.01, Noldus Information Technology). In total we obtained 20 hours of video-recordings from each experiment. We focused on the behaviour of all ants present on the artificial hot spot and recorded the time when a single ant entered and left the hot spot. From those data we measured the duration of each visit as a separate event, and also counted the total number of hot spot visits per day. We additionally quantified the total cumulative time all ants had spent sun basking on a given day and over the complete duration of the experiment. The maximal duration of sun basking in a complete experiment, i.e., for those ants that hypothetically expose themselves to the hot spot all time it was available, would be 20 hours (2 hours per day, over 10 days).

In experiment 1 we were not able to individually recognize workers because individual colour codes appeared in false colours under the IR lamp. Therefore, their correspondence with an ID table in the videos with quick moving ants was not possible. The total number of sun-basking ants in consecutive days of the experiment was obtained from daily counts at the end of the sun-basking period, after the marking. With the counts of the total number of hot spot visits, the cumulative duration of the hot spot visits per each day, and the number of sun-basking ants per each day, we could estimate the number of hot spot visits for one ant and also the average duration of sun basking of one visit to the hot spot.

Thanks to the individual marking using paper tags in experiments 2 and 3, all those parameters could be calculated separately for each individually marked ant. Ants that lost their tags were not considered. Continuous video recordings were evaluated only for experiment 1 and experiment 2 to enable comparison of results obtained with the two different marking methods (see part 2.3.). In experiment 3 only the presence or absence of individually marked ants observed on the hot spot on each experimental day was scored.

### 2.5. Respiration rates as a function of temperature

**Fig 4:**
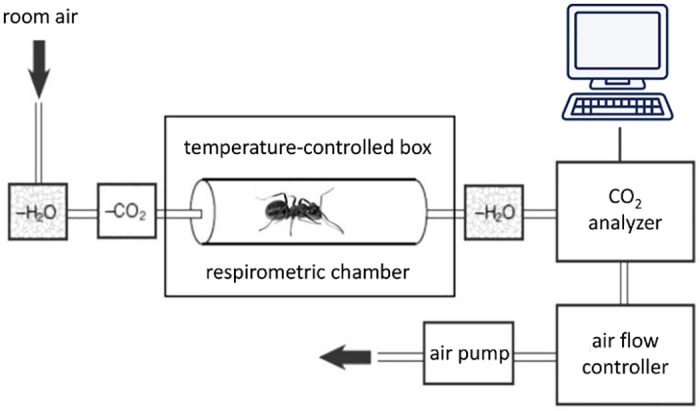
Experimental setup for respirometric measurements. Adapted from [32]; description in the text.

Respiration rates of single ants were measured with flow–through respirometry (Sable Systems), using a setup previously described in [32]. Briefly, CO_2_- and H_2_O-free air was drawn through an acrylic respirometric chamber (5.2cm x 2.8cm x 2.8cm; volume = 40.8cm^3^) at a flow rate of 300ml*min^–1^ STP controlled by a mass-flow controller. The CO_2_ produced by a single ant was measured by an IR-CO_2_ analyzer (LI-COR CO_2_ analyzer LI-6251, LI-COR Inc., Lincoln, NE, USA). To quantify the relationship between respiration rates and temperature, the respirometric chamber and connecting pipes were enclosed in a temperature-controlled box (Fig 4), using a Peltier-effect constant temperature system (Sable Systems, PTC-1-W). Our aim was to quantify the CO_2_-production rate for *F*. *polyctena* ants as a function of temperature, starting from 5°C, which is the low temperature limit of ant activity, up to 35°C, which is the highest temperature limit [33–35]. Measurements of respiration rates in individual workers were performed at 5, 10, 20, 30 and 35°C.

Prior to the measurements, ants were allowed to acclimatize at the temperature to be assayed, to quantify the effects of a long-term exposure and so to characterize the temperature-dependence of respiration across the ranges of temperatures ants experience after overwintering. A group of approx. 30 ants was kept in a thermostatic box with the target temperature for 1 week. After at least three days of exposure to the target temperature, respirometric measurements were performed on 12 ants individually. Only 9 ants were measured at the temperature 35°C because of high mortality.

The same group of animals was used for all temperatures assayed, i.e., single ants experienced the step changes from 5 to 35°C over 5 weeks, with one week at each target temperature. This method was used to simulate the natural pattern of temperature increase in early spring, after overwintering, and also to avoid heat-shock reactions. The total duration of the experimental period was 5 weeks in total, during which ants were provided daily with *ad libitum* water and honey-water solution.

### 2.6. Respirometry of sun-basking and non-sun-basking ants

The measurements were aimed at comparing the respiration rates of non-sun-basking workers with those of sun-basking workers before and two days after they were allowed to expose themselves to a heat source over 10 days, to highlight a putative long-lasting increase in respiration rates triggered by sun basking. Prior to each sun-basking experiment, the respiration rate of single ant workers from the subcolony was measured as CO_2_-production rates using flow-through respirometry (Fig 4). Contrary to previous, pioneer respirometric measurements on *F*. *polyctena* wood ants [22], we were able to measure respiration rates of single individuals for the first time, and thus to control for possible group effects [36]. Respiration of single ants was measured for 7 minutes to allow reliable measurements and to avoid stressful conditions because of the confinement in the respirometric chamber. Levels of activity of individual ants inside the chamber were scored as percentage of time with active movements from the total time spent in the chamber.

Before each sun-basking experiment, the respiration rate of 10 individual ants was measured. At the end of the experiment in which sun-basking workers were allowed to expose themselves to the heat source at self-determined intervals over the 10 days, respiratory measurements of single individuals were additionally performed in 20 workers, 10 sun-basking and 10 non-sun-basking workers. Measurements were done two days after long-lasting exposure to the heat source to quantify truly sustained increases in respiration rates different from short-term, transient changes that may occur readily after exposure. A total of 90 ants were measured (3 experiments, 30 ants each) and weighed. All measurements were performed at a temperature of 10°C, the same temperature ants were kept at, to allow comparisons.

After the end of each sun-basking experiment, ants were individually weighed to the nearest 0.01 mg with a laboratory scale (Kern ABT 120-5DM). A total of 140 ants were weighed, both sun-basking and non-sun-basking ants from all three experiments. For further calculations, the body mass of ants from experiment 1 (colour marking) and experiments 2 and 3 (paper tags) was handled separately because of the added weight of the paper tags (aprox. 0.5mg). Size of ants that died during the experiments was measured as maximal head-width in a frontal view. Dead ants were decapitated and head widths in a line below eyes were measured from microscope pictures. A total of 64 ant heads, both of sun-basking and non-sun-basking ants from all three experiments, were measured. Dead ants were not weighed because of their water losses, and we never killed and decapitated living workers for measurements.

### 2.7. Statistics

All statistical computations were performed using the “R” program (2.15.2 version) [37]. According to behavioural differences observed in the sun-basking experiments, we classified *F*. *polyctena* ants as sun-basking workers, i.e., those ants that visited the hot spot, and non-sun-basking workers, i.e., those ants that did not. Data used for calculations are accessible as Supplementary Material (S1 Table).

Mortality of sun-basking and non-sun-basking workers per day was compared by paired t-tests (data were normally distributed after a test for normality). A set of several separate t-tests (non paired two-tailed) was performed to compare behaviour (time spent sun basking, number of visits), body size (head width, body mass) and respiration rates (before and after sun basking) between the two groups. For comparisons of body sizes, the Welch correction was used because the standard deviation of the two tested groups differed significantly. Respiration rates of ants at different temperatures were compared also by an unpaired two-tailed t-test. An independent t-test was performed for each comparison.

To evaluate which factors significantly affected ant behaviour, i.e., the number of ants present on the hot spot or the duration and the number of visits on a given experimental day, we performed multiple regression analyses with the following equations: “response variable ~ calendar date + day order in experiment (1–10) + average temperature of the hot spot + maximal temperature reached in the ant cluster + air temperature in the thermostatic box + number of ants present the same day on the hot spot + number of ants present the previous day on the hot spot”. For the final model we used only those variables that showed a significant effect and increased the proportion of explained variability in our data (best minimal model).

## 3. Results

### 3.1. General description of sun-basking behaviour under laboratory conditions

Under laboratory conditions, wood ants displayed sun-basking behaviour. A small subset of workers, separated from the queen and provided with nest material containing colony odours, performed normal activities, i.e., nest building, exploration and collection of dead crickets, honey-water solution and water, and even sun basking on the artificial hot spot. Thanks to individual marking we were able to follow individual ants and thus to reveal that only some workers repeatedly visited the artificial hot spot to sun bask, those we called sun-basking workers.

Even though all ants had free access to the outside arena before the sun-basking experiment, only a few ants were observed sun basking in the first days. The finding of the hot spot probably happened accidentally during the exploration trips. Once the hot spot was found, however, we observed excited behaviour among ants (increased number of contacts, antennation, rapid movements of single ants in between others) and also increased traffic between the nest and the hot spot, which resulted in a subsequent large number of ants visiting the hot spot that gradually increased during the whole experimental period.

From our observations it seems that foragers and sun-basking workers acted independently from each other. The hot spot was located at the very opposite corner of the location of food and water, i.e., foraging ants usually went directly to the food source, collected some honey water or crickets, and then returned to the nest. Behaviour of sun-basking ants was different. They usually emerged from nest individually and clustered on the hot spot in large numbers (Fig 2). A stable cluster of sun-basking ants appeared to stay on the hot spot as long as the heat source was provided, even though the individual workers did not stay long.

There was a permanent movement inside the cluster as previously described [29], with individual ants often changing their position in the cluster, some departing from it and some others joining (see S1 Video in the Supplementary Materials). Ants departing from the cluster did not always go directly to the nest; often they went to the water source and then returned to the hot spot. Occasionally, we observed a single ant going from the nest to the sunning cluster, grabbing one of the sun-basking ants by its leg or antennae, and pulling it back to the nest. Twice we also observed an ant taking one of the sun-basking ants and transporting it in pupal position back to the nest. The reason for this behaviour is unclear.

### 3.2. Experiment 1: Colour marking of ants engaging in sun basking

**Fig 5:**
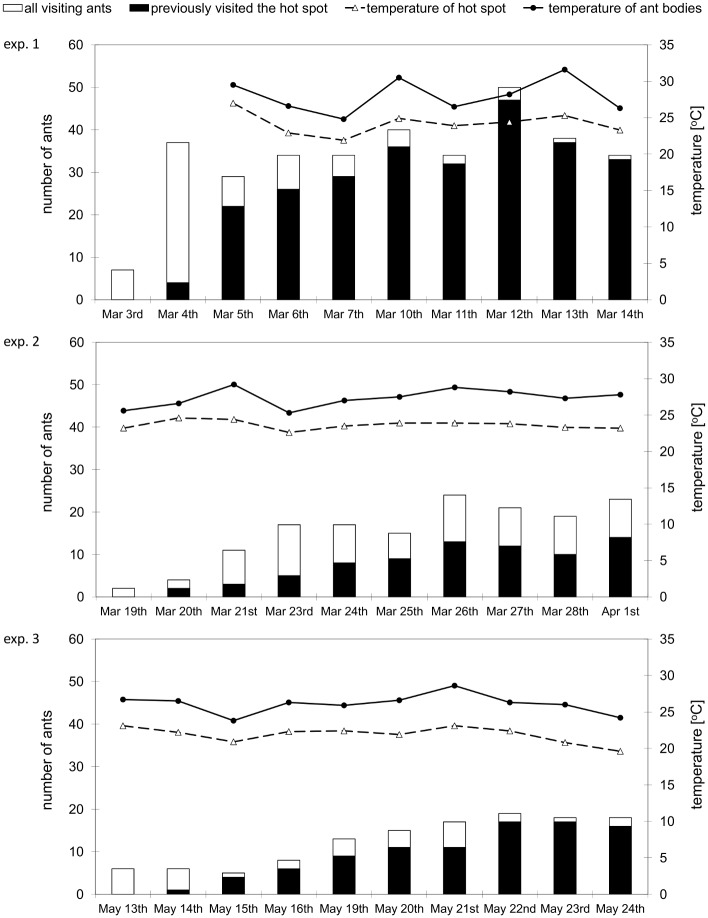
Results of the three sun-basking experiments. Both the total number of ants observed sun-basking for each day of the experiment and the number of ants observed sun-basking on the previous days are depicted. Note that in experiments 2 and 3 (ID tags), the total number of ants includes both ants with the ID tag and those without the tag (unidentified individuals). Average temperature of the hot spot and maximal temperature of the sun-basking cluster obtained by the IR camera are also depicted.

Our results from the colour marking experiment showed that not all ants from a subcolony of *F*. *polyctena* took part in sun basking. Some ants stayed in the nest for the whole time of the experiment and were never observed outside. They were therefore called non-sun-basking ants and were not marked. Other ants were often observed on the hot spot and therefore marked with colours; those we called sun-basking ants. The sun-basking ants made up 71% of the subcolony in experiment 1. The frequency of visits to the hot spot differed among sun-basking ants. Some ants repeatedly visited the hot spot, i.e., they were marked with several different colours on a daily basis, and some others sun basked only once (Fig 5). Daily mortality of sun-basking ants did not differ from mortality of non-sun-basking ants (paired two-tailed t-test, p = 0.1486, DF = 29, t = 1.484) and no pattern in mortality appeared between the consecutive days of the experiment.

Although we were not able to distinguish ant workers individually in this experiment, we could estimate from our video recordings the duration of one visit to the hot spot for an “average ant”. For each day of the experiment, we counted from 108 to 720 visits to the artificial hot spot and recorded their duration in the Observer program (without knowing the identity of the ant). We counted from 7 to 50 sun-basking ants per one day, with the number of sun-basking ants gradually increasing during the consecutive days of experiment.

For each ant we estimated the number of hot spot visits per one day and the sun-basking time per one day (see part 2.4) from the total number of hot spot visits and the total time (cumulative duration) of all hot spot visits on the given day (Table 1). For example, on the very first day of experiment 1 we counted 108 visits to the hot spot with a total duration of sun basking of 4 hours 56 minutes and 6 seconds. On this day there were only 7 ants sun basking, which resulted in an average of 15 visits and an average duration of the hot spot visit (time spent sun basking) equal to 42 minutes and 18 seconds for one ant. We also estimated the average duration of one hot spot visit, which varied over the days from 1 minute and 56 seconds to 7 minutes and 33 seconds. Both the number of hot spot visits and the time spent sun basking by one ant were quite variable over the days of the experiment too (Table 1).

**Table 1:** Daily results of the sun-basking experiments 1 and 2. The total number of hot spot visits recorded for each experimental day (total N visits), the cumulative duration of all hot spot visits and the total number of ants observed sun basking (N ants) on a given day are depicted. The second part of the table shows the estimated values for one ant and one visit. Information about time (cumulative duration, time spent per 1 ant, duration of 1 visit) is given as hours:minutes:seconds (h:min:sec). Note that data from experiment 2 are only from those ants whose ID tags remained and were therefore identifiable. Unidentified individuals were not included in the table.

day	date	N ants	cumulative duration (h:min:sec)	total N of visits	visits per 1 ant	time per 1 ant (h:min:sec)	duration of 1 visit (h:min:sec)
**Experiment 1**							
1	03.03.	7	4:56:06	108	15.43	0:42:18	0:02:45
2	04.03.	37	23:15:12	720	19.46	0:37:42	0:01:56
3	05.03.	29	17:42:10	499	17.21	0:36:38	0:02:08
4	06.03.	34	41:45:05	505	14.85	1:13:41	0:04:58
5	07.03.	34	25:56:57	471	13.85	0:45:48	0:03:18
6	10.03.	40	37:54:05	357	8.93	0:56:51	0:06:22
7	11.03.	34	43:59:05	544	16.00	1:17:37	0:04:51
8	12.03.	50	39:56:28	508	10.16	0:47:56	0:04:43
9	13.03.	38	41:06:19	327	8.61	1:04:54	0:07:33
10	14.03.	34	37:07:55	394	11.59	1:05:32	0:05:39
**Experiment 2**							
1	19.03.	2	2:40:56	54	27.00	1:20:28	0:02:59
2	20.03.	4	3:57:56	50	12.50	0:59:29	0:04:46
3	21.03.	11	6:55:09	93	11.63	0:51:54	0:04:28
4	23.03.	17	13:55:52	277	18.47	0:55:43	0:03:01
5	24.03.	17	10:03:36	160	10.67	0:40:14	0:03:46
6	25.03.	15	13:28:11	90	6.43	0:57:44	0:08:59
7	26.03.	24	25:19:32	76	4.22	1:24:25	0:20:00
8	27.03.	21	18:51:02	161	10.73	1:15:24	0:07:02
9	28.03.	19	13:40:20	95	6.79	0:58:36	0:08:38
10	01.04.	23	33:08:26	272	11.83	1:26:27	0:07:19

### 3.3. Experiments 2 and 3: Individual tagging with ID tags

The results of experiments 2 and 3, in which ants were individually marked with paper ID tags, confirmed our observations from experiment 1. Again, we observed sun-basking ants and non-sun-basking ants in the laboratory subcolony of *F*. *polyctena* and found increasing numbers of ants visiting the hot spot over the 10 days of the experiments, and increasing number of ants that repeatedly visited the hot spot (Fig 5). However, the proportion of sun-basking ants and non-sun-basking ants was inverse as compared to that found in experiment 1, with only 33% of sun-basking ants.

The daily dynamics of sun-basking behaviour observed in experiment 2 was similar to that observed in experiment 1. Both the total number of visits per day and the total number of ants observed sun basking increased during the experiment; however, compared to experiment 1 it was nearly twice lower (Table 1). We recorded from 50 to 277 visits to the hot spot per day and counted from 2 to 24 sun-basking ants per day in experiment 2. Such low numbers corresponded to the lower proportion of sun-basking ants observed in this experiment.

Mortality was higher (total mortality 41 and 27 ants out of 100 ants, for experiments 2 and 3) as compared to the previous experiment (mortality 18 ants for experiment 1), probably due to the different marking techniques. There occurred also a substantial loss of paper tags, which resulted in a lower number of ants that could be identified at the end of experiments.

**Fig 6:**
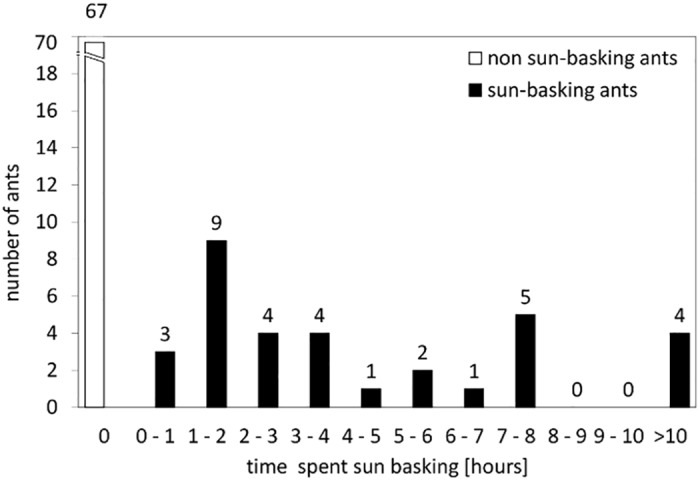
Behaviour of individual sun-basking ants. Histogram of the total cumulative time spent by non-sun-basking and by sun-basking workers during the whole duration of experiment 2. Note the last category with more than 10 hours of sun basking.

Thanks to the ID tags we were able to identify individual ants in the digital video recordings and to count both the number of visits and the total time spent sun basking for each individual worker. Our results clearly showed that there was a large variability in sun-basking behaviour among individual workers. Some ants visited the hot spot more often than others did. Most ants spent between less than 1 up to 4 hours, while some of them spent more than 10 hours sun basking over the 10 days of the experiments, from the maximal 20 hours of video-recording (Fig 6).

In experiment 2 we recorded 33 sun-basking ants in the subcolony counting 100 ant workers. The sun-basking ants performed a total of 1 to 139 visits to the hot spot, with an average number of hot spots visits equal to 40. The distribution of hot spot visits was positively skewed. Approximately, only one third of the sun-basking ants (12 ants) visited the hot spot more often than the average visit rate, those we called “frequent visitors”. They visited the hot spot in more than 5 out of the total 10 days of the experiments, with a significantly higher average number of visits than those of regular visitors (73.0 visits/ant as compared to 21.9 visits/ant; unpaired two-tailed t-test, t = 5.495, DF = 31, p<0.001). Only seven workers were responsible for half of all observed visits. In addition, the frequent visitors spent significantly longer average time sun basking than the regular visitors (8.2 hours/ant as compared to 2.2 hours/ant, from the total 20 hours of video-recording; unpaired two-tailed t-test, t = 11.416, DF = 31, p<0.001).

**Fig 7:**
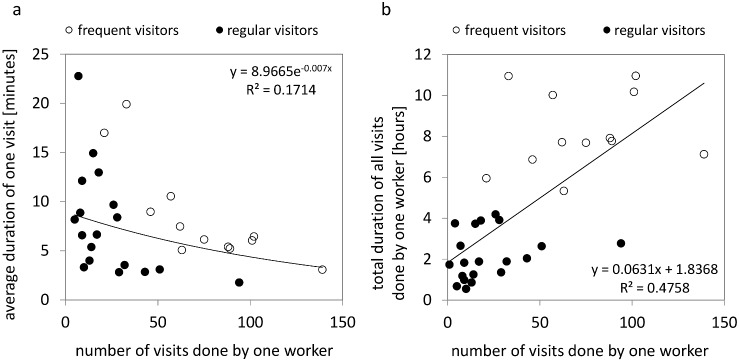
Duration of one visit and total duration of all hot spot visits for individual ant workers. (a) Relationship between the number of visits to the hot spot and the average duration of one visit, for individual ants. The regression line did not include the two outliers described in the text. (b) Relationship between the total number of visits to the hot spot and the total time spent sun basking (as cumulative duration of all visits), for individual ants. Line and equation for the exponential (a) and the linear regression (b) are provided; both are statistically significant at p<0.05.

The longest duration of a single sun-basking visit was 104.3 minutes. However, in this and in a second extreme case (56.3 mins) we cannot exclude the possibility that they are caused by a mistake during video evaluation, either because the leaving ant was not properly identified, or the corresponding time counter was not stopped in the Observer program. Both of these outliers represent ants with a low number of visits. If these outliers are removed from the analysis, the duration of one visit varied from 1.8 minutes to 22.8 minutes (9.5 minutes in average). The duration of one visit significantly decreased with increasing number of visits (Fig 7a).

The total cumulative time all ants spent sun basking in the whole experiment varied from 33 minutes to 10 hours 57 minutes. The average length of sun basking for one ant in the whole experiment was 4 hours 36 minutes. Again, the distribution of sun-basking times was positively skewed; approximately one third of the sun-basking ants (12 ants) spent more time sun basking than the average time. The total time spent sun basking by individual ants increased linearly with the number of visits performed by those ants (Fig 7b), but showed no significant correlation with the average duration of the individual visits performed by these ants (r = -0.103, ns). This suggests that the total cumulative time spent sun basking was driven by the number of visits rather than by the duration of the individual visits.

### 3.4. Factors affecting ant behaviour

Since the number of ants on the hot spot differed between experiments, we investigated the extent to which the number of ants correlated with experimental variables such as date, day order in the experiment (1 till 10), air temperature in the thermostatic box, average temperature of the hot spot, maximal temperature of the ant cluster, and presence of ants on both the experimental day and on the previous day.

We found that both the number of ants that took part in sun basking and the number of visits to the hot spot negatively correlated with the date of the three experiments. The opposite was true for the successive days in a given experiment: the later the day in the experiment, the more ants participated, and more visits occurred. Air temperature inside the box, which slightly oscillated around the 10°C established during the experiments (usually in less than 1°C, yet some unwanted larger variations down to 8.4° and up to 12.4°C occurred occasionally), affected both the number of ants and visits to the hot spot positively. The number of ants positively correlated with the maximal temperature reached in the sun-basking cluster.

According to the multiple regression model (Table 2), however, only the date and the day order in the experiment had a significant effect on the number of ants (p<0.0001 for both). The effects of other variables as air temperature were overlaid by the effect of date. On the contrary, the number of visits to the hot spot was significantly affected by the number of ants present on the day of the experiment, by the air temperature and by the maximal temperature of ant cluster (p<0.0001). Duration of one visit was significantly affected by both the air temperature and the maximal temperature of the ant cluster (p<0.001). The number of ants present on the day of the experiment strongly correlated with the number of ants present on the previous days of the experiment (R = 0.80), leading to a cumulative effect. The number of ants present on the day of the experiment, as well as on the previous day, had a strong positive effect on the number of hot spot visits too.

**Table 2:** Results of multiple regression model. Best minimal models for number of ants performing sun-basking behaviour, number of visits to the hot spot and duration of one visit are depicted. “Ex.day.o” for day order in the experiment (1–10), “t.air” for air temperature of in the thermostatic box, “t.hot spot” for average temperature of the hot spot, “max.cluster” for the maximal temperature of the ant cluster, “n.ants” for the number of ants present on the experimental day.

factor	DF	sum Sq	F value	p
**Number of ants performing sun-basking behaviour**				
model: R^2 =^ 0.76, F-statistic: 26.5 on 2 and 17 DF				
Date	1	1 684	36.0	<0.001
ex.day.o	1	794	16.9	<0.001
residuals	17	794		
**Number of visits to the hot spot**				
model: R^2 =^ 0.92, F-statistic 57.14 on 3 and 14 DF				
n.ants	1	382 988	127.5	<0.001
max.cluster	1	71 176	23.7	<0.001
t.air	1	60 561	20.17	<0.001
residuals	14	42 040		
**Duration of one visit**				
model: R^2 =^ 0.61, F-statistic 11.75 on 2 and 15 DF				
t.air	1	98	14.0	<0.01
t.cluster	1	66	9.5	<0.01
residuals	15	15		

### 3.5. Respiration rates of single workers as a function of temperature

We measured respiration rates of *F*. *polyctena* workers (CO_2_ production rates in microliters per hour) in the temperature range that wood ants encounter in nature, from 5°C to 35°C [33–35], to quantify the effects of a long-term exposure and so to characterize the temperature-dependence of respiration across the ranges of temperatures ants experience after overwintering. The range includes the maximal temperatures of sun-basking ants we measured in our experiments using the artificial hot spot, which varied between 23.8 and 31.6°C. However, sun-basking ants experience a rather quick increase of body temperature during exposure, which may lead to a temporary-increased respiration rate as compared to those measured after a long-term exposure to a constant temperature.

As a consequence, the measurements were aimed at quantifying the steady-state respiration rates as a function of temperature, since ants were first allowed to acclimatize at the temperature to be assayed. The respiration rate of a single wood ant worker between 5 and 10°C was lower than 10 μl CO_2_*hour^-1^ and it did not differ significantly between the two temperatures (two-tailed, unpaired t-test, p = 0.345, DF = 26, t = 0.962). As expected for ectothermic animals, the respiration rates increased with increasing temperatures. The levels of locomotor activity also increased with increasing temperatures: while at 5°C ants spent 65% of time sitting still, at 35°C it was only 9%.

**Fig 8:**
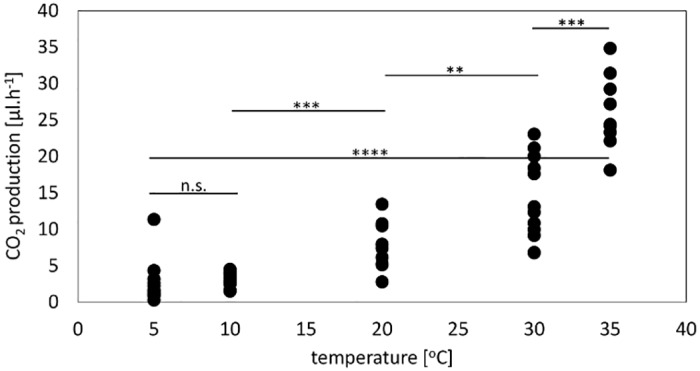
Respiration rates of single *F*. *polyctena* workers as a function of temperature, after long-term exposure. The respiration rate was measured individually at 5, 10, 20, 30 and 35°C. Significant differences between the assayed temperatures are marked with an asterisk (** = p<0.01, *** = p<0.001, **** = p<0.0001, n.s. = non significant)

In the whole range of the measured temperatures (5–35°C), the respiration rates of ant workers increased 10 times, from 2.27 to 26.09 μl*hour^-1^ in average (Fig 8). Minimal and maximal values recorded were 0.28 and 34.83 μl*hour^-1^ at 5 and 35°C, respectively. At temperatures higher than 10°C, all comparisons (10 vs. 20, 20 vs. 30, 30 vs. 35°C) provided significant differences in respiration rates (two-tailed unpaired t-test, for 10 to 20°C p<0.0001, t = 5.02, DF = 22; for 20 to 30°C p<0.005, t = 3.25, DF = 21; for 30 to 35°C p<0.0001, t = 4.97, DF = 19) (Fig 8). Comparison of respiration rates at 5 to 35°C yielded also highly significant differences (two-tailed, unpaired t-test p<0.0001, DF = 23, t = 15.512).

In the temperature range experienced during sun basking in our experiments (minimal air temperature 8.4°C and maximal temperature of the sun-basking cluster 31.6°C), the steady-state respiration rates of workers varied between 1.49 and 23.05 μl*hour^-1^. Based on our measurements over a wide range of temperatures, mean respiration rates at a body temperature equal to 10°C averaged 3.03 μl*hour^-1^, 7.52 μl*hour^-1^ at a body temperature equal to 20°C, and 14.1 μl*hour^-1^ at a body temperature equal to 30°C. This means that an increase of body temperature from 10 to 20°C and from 10 to 30°C causes a 2.5-fold and a 4.6-fold increase in respiration rates, respectively.

**Fig 9:**
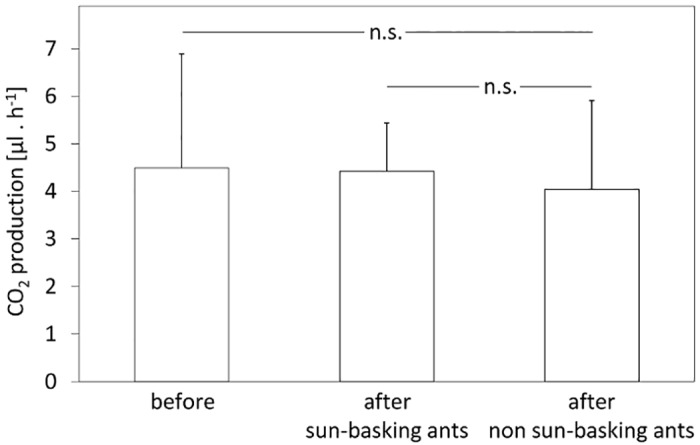
Respiration rate of workers before and after sun basking. The bars show respiration of single *F*. *polyctena* workers (CO_2_ production in microliters per hour) measured at 10°C, before and two days after a 10-days-period of self-determined sun basking, for both sun-basking ants and non-sun-basking ants. Mean and standard deviation are given. Differences between the compared groups are not statistically significant (p>0.05) in all cases.

To investigate whether the sun basking led to a sustained high metabolic rate consistent with the expected increased catabolism of lipid reserves, we measured and compared respiration rates of non-sun-basking workers with those of sun-basking workers before and two days after they were allowed to expose themselves to a heat source over 10 days, at self-determined intervals. Surprisingly, there was no difference in the respiration rate of ants before sun basking and two days after the 10-days-period of autonomous sun basking (two-tailed, unpaired t-test, p = 0.8868, DF = 53, t = 0.1430) (Fig 9). Sun-basking and non-sun-basking ants showed also no differences in their standard respiration rates (two-tailed unpaired t-test, p = 0.3657, DF = 50, t = 0.9128).

Over the 10 days of the experiments, those individual ants that were taken for the respirometric measurements visited the hot spot repeatedly, for a period that varied between 4 and 8 days (n = 26). The self-determined time spent sun basking by individual ants over that period averaged 8.3 hours, from a maximal time of 20h video-recording time over the 10 days. Since we only video-recorded one half of the maximal time of potential exposure, the estimated total time of sun basking would therefore average 16.6 hours over the ten days.

We additionally checked for possible morphological differences between sun-basking and non-sun-basking ants, and found that neither head width (unpaired t-test, p = 0.6188, DF = 62, t = 0.5001) nor fresh body mass, considered separately for experiment 1 (unpaired t-test, Welch correction, p = 0.1661, DF = 38, t = 1.412) and experiments 2 and 3 (unpaired t-test, Welch correction, p = 0.0797, DF = 58, t = 1.783), differed between them.

## 4. Discussion

In our laboratory experiments, we succeeded in triggering sun-basking and clustering behaviours in *F*. *polyctena* workers on an artificial hot spot provided by an infrared source, as previously described by [11, 29]. Sun-basking behaviour was similar to that described under field conditions, which is assumed to serve as one of the mechanisms underlying nest heating in early spring [2, 3, 15, 24]. Our results go beyond previous studies on sun basking in wood ants [11, 29] by describing the behaviour of individually-marked workers over several days.

Results indicated that only about one third of all ants in the experimental subcolonies engaged in sun-basking behaviour by repeatedly visiting the hot spot, suggesting that sun basking may be a task only performed by a subset of workers. Task allocation in social insects is a very common phenomenon, often dependent on age or size [38–40]. Interestingly, sun-basking and non-sun-basking ants did not differ in head width, body mass, nor in their standardized respiratory rates, indicating that so far unknown physiological differences between them may underlie the observed differences in behaviour.

Among those workers that performed sun-basking behaviour, there was a large variation in the number of visits to the hot spot. Since the variability among workers in the average visit duration was rather small, the number of visits largely determined the total time spent sun basking and consequently the total amount of thermal energy individual ants can accumulate and eventually carry into the nest. The number of visits was strongly positively skewed, with most workers being non-sun-basking ants. Even among sun-basking ants, most workers visited the hot spot just once or only a few times, while most visits were performed by only a few workers. This suggests that the threshold that triggers sun-basking behaviour may be highly variable within a colony, as shown, for instance, in thermoregulatory behaviours of bumblebees [41].

The observation that the duration of visits varied less than the number of visits may result from the fact that sun-basking ants may approach, after a certain period of time on the hot spot, a lethal temperature that may limit the maximal duration of their exposure. However, it is likely that the variability in visit times under natural conditions may be larger than in our experiments due to the large variation of incoming solar energy as a result of whether fluctuations.

Similar to field observations, sun-basking clusters in laboratory subcolonies of *F*. *polyctena* occurred most often in early spring [4, 24]. The total number of sun-basking ants decreased over time in the three replicates of our experiments. We recorded the highest fraction of sun-basking ants (71%) in the very first experiment (at the beginning of March), whereas in the next two experiments (end of March and middle of May), the proportion of sun-basking ants decreased to 33 and 21%, respectively. The reason for this decline in sun-basking behaviour is most probably its relationship with the timing of brood development [24]. Under natural conditions, ant workers are expected to perform sun basking as a thermoregulatory behaviour only when sexual brood is present in the nest, i.e., in early spring [11, 16, 17]. Yet we cannot completely rule out the effects of increased mortality and losses of paper tags we experienced in our experiments 2 and 3, which resulted in a lower number of ants that could be identified as sun-basking ants at the end of experiments.

It has been argued that sun basking is more likely performed by the youngest workers in the nest, which often accumulate large amount of lipids and are therefore defined as repletes [11, 27]. A number of workers may never directly participate in sun basking, because they may indirectly be activated over the early spring months through the continuous increase in the mean nest temperature [24, 27]. Since we did not measure lipid contents in sun-basking and non-sun-basking workers, it remains as an open question whether the probability to engage in sun basking correlates with the lipid contents of the workers.

It is tempting to hypothesize that the proper timing of sun-basking behaviour in workers may be determined by an annual rhythm controlled by an endogenous clock. Such an endogenous clock that drives the timing of egg lying is already known in red wood ant queens [16]. Some physiological changes in workers, mainly the accumulation of lipid reserves, appear to be also controlled by annual clocks, since they even occur at high temperatures and long photoperiods [24, 42]. We suppose that in our last experiment in May, the proper period to stimulate sun-basking behaviour in workers was already over, even though they were kept in the laboratory at a stable temperature (10°C) and unchanged photophase length. The observed significant effects of both date and day order on the number of ants performing sun-basking behaviour support this assumption.

According to our results, clustering behaviour in red wood ants is also affected by air and hot spot temperature, which positively affects both the number of visits to the hot spot and the duration of one hot spot visit. This is in agreement with previous results showing that both the probability of occurrence and the number of workers showing sun-basking behaviour increased with increasing temperature on the hot spot, and also with decreasing outside temperature [29]. From the results of own preliminary experiments, there appears to be a minimum threshold temperature on the hot spot that needs to be exceeded to stimulate workers to cluster on the spot. At temperatures on the hot spot below 20°C, almost no sun-basking behaviour occurred.

In a bee hive [43–46] or in bumble bee nest [43, 47], nest heating largely relies on metabolic heat produced by the workers’ flying muscles. Ant workers lack flying muscles, yet it is supposed that metabolic heat production by workers represents an important part of the total heat generated in a wood ant nest, together with the contribution of the microorganisms/decaying nest material [2, 3, 11, 13, 14]. Calorimetric measurements in one laboratory colony indicated that workers are responsible for more than 80% of the total heat production when the nest material is wet [13]. In addition, results of a recent field study on colony respiration of a wood ant nest also support the view that the ants’ contribution to the overall respiration of a nest is much higher than that of the nest material [14].

According to our and other authors´ results [24, 25, 29], ants exposing themselves to solar radiation increase their body temperature, which has been hypothesized to activate lipid catabolism in reserve workers and to lead to a sustained, long-term increase of respiratory rates and associated higher production of catabolic heat [11]. The temperatures experienced during sun basking in our experiments varied between 23.8 and 31.6°C. Our measurements of individual respiration rates as a function of temperature confirmed that high body temperatures caused a significant increase in respiration rates, not surprisingly for ectothermic animals. The rates measured at a body temperature equal to 31.6°C were five times larger than those measured at a body temperature of 10°C. We can expect even higher metabolic rates in the short term, since body temperatures exceeding 40°C were measured in sun-basking workers of *F*. *polyctena* under natural colonies (Kadochová & Frouz, unpublished results).

We measured respiration rates of red wood workers in a temperature range going from 5°C to 35°C, which are approximately the limits of wood ant activity in nature [33–35]. At low temperatures, between 5 and 10°C, respiration rates were low, and significantly increased with increasing temperatures. This is in a good agreement with early results showing an exponential increase in the CO_2_ production rate of wood ants up to 40°C, using a Warburg-manometric respirometer [22]. In the range between 5 and 35°C, respiration rates also increased 10 times in average. The agreement between Kneitz´s and the present results is very interesting, since we measured respiration of single workers whereas he measured respiration of a group of 4 workers [22], so we can conclude that there is no group effect [36] concerning metabolic rate in *F*. *polyctena* ants.

However, higher respiration rates of *F*. *polyctena* workers as expected to be achieved during sun basking did not remain high in the long term, as measured two days after sun-basking workers were allowed to expose themselves, at self-determined time intervals, to a heat source over 10 days. Even more striking, respiration rates of ants before and after sun-basking, measured at the same temperature, were similar. Therefore, the hypothesis that sun basking in wood ants serves as a trigger that starts an autocatalytic degradation of stored lipids and therefore leads to a sustained, long-lasting increase in respiratory rates [11, 26], finds no support.

It is an open question whether ants, over the short time of our laboratory experiments, changed the catabolized energy substrates, thus leading to a different interpretation of our indirect calorimetric measurements of CO_2_ production rates. If ants would have for instance changed from carbohydrate to lipid metabolism (after lipid mobilization), the resulting change in the respiratory quotient (from 1 to 0.7) may have reduced the rate of CO_2_ production per unit of catabolized substrate, which could have been compensated by the hypothetical sustained increase in the respiratory rate. However, it appears unlikely that ants changed the catabolized energy substrates. In fact, wood ants were shown to catabolize both lipids and carbohydrates at comparable rates from the very beginning and over the complete period of sun basking, i.e., the months of early spring, and the absolute lipid amounts stored by single workers were 8 to 9 times larger than those of carbohydrates [26].

Interestingly, early measurements [22] showed that the mass-specific respiration rates of replete workers were lower than those of “external” workers, which had no large lipid reserves. In addition, the respiration rates of replete workers were significantly lower in winter, thus suggesting a sustained activation of metabolism in the summer months. In our opinion, whether replete workers have indeed low respiration rates in winter needs to be confirmed and explored in more detail in future studies. Their lower respiration rates may have resulted from both their reduced locomotion inside the respirometric chamber, as noticed in the study [22], and the higher proportion of less metabolically active tissue like fat, which may comprise up to 20% of the workers’ fresh body mass [26].

Thus, we found no evidence for a physiological activation leading to a sustained high metabolic rate in sun-basking ants, which could contribute as one of the heat sources responsible for the increase of temperature in *F*. *polyctena* nests in early spring. We speculate, based on our observations with video thermography and as previously suggested [2–4, 15, 24, 25], that heat transported by sun-basking ants into the nest may contribute to nest heating, together with other internal heat sources like catabolic heat produced by workers and by the nest material, as discussed above.

The idea that heat can be transported by workers into the nest, as originally formulated [24] and further suggested by a number of authors [2–4, 15], offers an explanation for the adaptive value of sun-basking behaviour. As indicated in the Introduction, it has been hypothesised that workers sun basking on the nest surface in early spring can effectively absorb solar radiation in their bodies and, thanks to the high thermal capacity of the haemolymph, carry the heat into the nest where it dissipates [24]. Warm conditions inside the nest enable faster development of both worker and sexual brood [16, 38], thus increasing the colony fitness. The results from a recent study in our laboratory [25], and also the observations with the help of thermographic images from the present study (Fig 2 and S1 Video), indicate that heat carriage by sun-basking workers indeed occurs.

More detailed studies and calorimetric measurement are still needed to show whether ants are capable of carrying heat amounts into the nest sufficient to explain early nest heating. To estimate the amount of heat potentially carried into the nest by a large group of sun-basking ants in nature, we need to know the cooling rate and a the heat capacity of the ant bodies, which were so far not reported in the literature. Population size of red wood ant colonies may reach millions [3, 4, 12], thus the amount of heat that could be temporarily stored in the ant bodies during sun basking and carried into the nest might be huge. By this massive indirect income of solar energy, the quick increase of inner nest temperature observed in red wood ants by several authors [2, 3, 15] could be explained.

## Conclusion

There are sun-basking ants and non-sun-basking ants in colonies of wood ants; the fraction of sun-basking ants in a colony decreases over time. Among sun-basking ants, some individuals spend significantly more time sun basking and perform more visits to the hot spot than others. Sun-basking ants and non-sun-basking ants do not differ in body size nor in standardized metabolic rates. There are no differences in the respiration rate of ants before sun basking and two days after they are allowed to expose themselves to a heat source over 10 days, at self-determined intervals. Therefore, the transient increase in the workers’ body temperatures and, as a consequence, in their respiration rate during sun basking does not lead to a sustained, long-term increase in respiration rate consistent with a higher rate of lipid catabolism. Based on our measurements, we argue that self-heating of the nest mound in early spring has therefore to rely on alternative heat sources, and speculate that physical transport of heat in the ant bodies may have a significant effect.

## Supporting Information

S1 Table Data used for calculations and figures in the manuscript. Data for each figure are depicted in a separate sheet.
(XLSX)

S1 Video Supplementary Material. Thermographic video showing behaviour of ants visiting a hot spot. A hot spot with a cluster of sun-basking ants is visible in the upper left corner (yellow-orange spot), which was generated with an IR lamp in a laboratory setting. In the upper and lower corners on the right, a vessel with water and a food patch are visible (both cold, depicted in dark blue). Two warm ant workers (orange) emerge from the cluster (time 00:00:01:30). They are heading down to the nest entrance (in lower part of screen) and spent some time exploring the bridge leading to the nest entrance (time 00:00:13:50). One of these workers enters the nest (time 00:00:37:05), while the other turns back and return to the hot spot after a while (time 00:01:01:23), joining the ants in the sun-basking cluster (time 00:01:07:15). On the top, a cold ant moves towards the hot spot (time 00:00:42:00), walks around and remains at the cluster, reaching a higher body temperature (00:01:01:00). Please note that ants leaving the hot spot slowly cool down over time, since the false colour is changing from bright orange to red and pink, which represents a temperature change from 23.5 to 21°C. The ants’ gaster cools down slower than other body parts. The thermographic video was taken during preliminary trials performed at *ca*. 15 ± 1°C ambient temperature. For the experiments presented in the manuscript, only long-term digital videos were used because the tags used to individually mark ants cannot be recognized on thermographic videos, and ambient temperatures were set at 10°C.
(WMV)
